# Supplementary material for: The Chinese version of the Maltreatment and Abuse Chronology of Exposure (MACE) scale: Psychometric properties in a sample of young adults
Source: PLoS One. 2022 Jun 30;17(6):e0270709. doi: 10.1371/journal.pone.0270709 (PMC9246159; doi:10.1371/journal.pone.0270709)
Supplement: S1 File — (DOCX) [file pone.0270709.s001.docx]

**Fig 1.** Rasch analysis of parental physical maltreatment subscale showing item characteristic curve, item information curve and test information function.

**Fig 2.** Rasch analysis of parental verbal abuse subscale showing item characteristic curve, item information curve and test information function.

**Fig 3.** Rasch analysis of non-verbal emotional abuse subscale showing item characteristic curve, item information curve and test information function.

**Fig 4.** Rasch analysis of peer emotional abuse subscale showing item characteristic curve, item information curve and test information function.

**Fig 5.** Rasch analysis of peer physical bullying subscale showing item characteristic curve, item information curve and test information function.

**Fig 6.** Rasch analysis of sexual abuse subscale showing item characteristic curve, item information curve and test information function.

**Fig 7.** Rasch analysis of witnessing violence to siblings subscale showing item characteristic curve, item information curve and test information function.

**Fig 8.** Rasch analysis of witnessing interparental violence subscale showing item characteristic curve, item information curve and test information function.

**Fig 9.** Rasch analysis of emotional neglect subscale showing item characteristic curve, item information curve and test information function.

**Fig 10.** Rasch analysis of physical neglect subscale showing item characteristic curve, item information curve and test information function.
