# Supplementary material for: The Chinese version of the Maltreatment and Abuse Chronology of Exposure (MACE) scale: Psychometric properties in a sample of young adults
Source: PLoS One. 2022 Jun 30;17(6):e0270709. doi: 10.1371/journal.pone.0270709 (PMC9246159; doi:10.1371/journal.pone.0270709)
Supplement: S2 File — (PDF) [file pone.0270709.s002.pdf]

中文版虐待年报量表

根据你在 1-18 岁期间的回忆，如果以下行为曾经发生在你的童年（十八岁以前）阶段，请勾选“曾发生”并尽量勾选这些伤害行为发生的所有年龄；如果没有发生过，请勾选“不曾发生”。

正式填写自此开始：

有时父母，养父母或其他与你住在一起的成人会对你做出具有伤害性的行为。如果以下行为曾经发生在你的童年（十八岁以前）阶段，请勾选“曾发生”并尽量估计这些伤害行为发生的所有年龄；如果没有发生过，请勾选“不曾发生”。、

1. 每年不止一次诅咒你、指责你，对你爆粗口，对你说“你真胖”、“你真丑”、“你真蠢”等等一些侮辱人的话。

曾发生	不曾发生

发生	1	2	3	4	5	6	7	8	9	10	11	12	13	14	15	16	17	18
年龄																		

2. 每年不止一次对你说伤人的话，致使你感到情绪糟糕、窘迫、羞辱。

曾发生	不曾发生

发生	1	2	3	4	5	6	7	8	9	10	11	12	13	14	15	16	17	18
年龄																		

3. 每年不止一次冲你大吼大叫。

曾发生	不曾发生

发生	1	2	3	4	5	6	7	8	9	10	11	12	13	14	15	16	17	18
年龄																		

4. 以让你害怕会遭受到身体伤害的方式行事。

曾发生	不曾发生

发生	1	2	3	4	5	6	7	8	9	10	11	12	13	14	15	16	17	18
年龄																		

5. 威胁要离开你或抛弃你。

曾发生	不曾发生

发生	1	2	3	4	5	6	7	8	9	10	11	12	13	14	15	16	17	18
年龄																		

6. 将你关在储藏室、阁楼、地下室、车库或者其它密闭空间里。

曾发生	不曾发生

发生	1	2	3	4	5	6	7	8	9	10	11	12	13	14	15	16	17	18
年龄																		

7. 故意推你、抓你、撞你、扇你耳光、掐你、用拳打你或用脚踢你。

曾发生	不曾发生

发生	1	2	3	4	5	6	7	8	9	10	11	12	13	14	15	16	17	18
年龄																		

8. 很用力地打你，让你留下的印记或伤痕好几分钟都没有消失。

曾发生	不曾发生

发生	1	2	3	4	5	6	7	8	9	10	11	12	13	14	15	16	17	18
年龄																		

9. 曾用力打你，或企图通过其他方式伤害你并且使你接受或需要接受药物治疗（吃药、擦药或者去医院等）。

曾发生	不曾发生

发生	1	2	3	4	5	6	7	8	9	10	11	12	13	14	15	16	17	18
年龄																		

10. 曾打过你的手臂、臀部或腿部。

曾发生	不曾发生

发生	1	2	3	4	5	6	7	8	9	10	11	12	13	14	15	16	17	18
年龄																		

11. 曾打过你裸露的臀部。

曾发生	不曾发生

发生	1	2	3	4	5	6	7	8	9	10	11	12	13	14	15	16	17	18
年龄																		

12. 曾用皮带、腰带、刷子、球拍、棒子等物品打过你。

曾发生	不曾发生

发生	1	2	3	4	5	6	7	8	9	10	11	12	13	14	15	16	17	18
年龄																		

13. 给你一些不合适的带有性色彩（如性挑逗、性暗示等）的评论或建议。

曾发生	不曾发生

发生	1	2	3	4	5	6	7	8	9	10	11	12	13	14	15	16	17	18
年龄																		

14. 以带有性色彩（如性挑逗、性暗示等）的方式触摸或爱抚你的身体。

曾发生	不曾发生

发生	1	2	3	4	5	6	7	8	9	10	11	12	13	14	15	16	17	18
年龄																		

有时父母，养父母或其他与你住在一起的成人会对你的兄弟姐妹（包括继兄弟姐妹）做出具有伤害性的行为。如果以下行为曾经发生在你的童年（十八岁以前）阶段，请勾选“曾发生”并尽量估计这些伤害行为发生的所有年龄；如果没有发生过，请勾选“不曾发生”。

15. 故意对你的兄弟姐妹（包括继兄弟姐妹）实施过推、抓、撞、扇耳光、掐、用拳打或用脚踢的行为。

曾发生	不曾发生

发生	1	2	3	4	5	6	7	8	9	10	11	12	13	14	15	16	17	18
年龄																		

16. 很用力地打你的兄弟姐妹（包括继兄弟姐妹)，让他们留下的印记或伤痕好几分钟都没有消失。

曾发生	不曾发生

发生	1	2	3	4	5	6	7	8	9	10	11	12	13	14	15	16	17	18
年龄																		

17. 用力打过你的兄弟姐妹（包括继兄弟姐妹)，或企图通过其他方式伤害他们并且使他们接受或需要接受药物治疗（吃药、擦药或者去医院等)。

曾发生	不曾发生

发生	1	2	3	4	5	6	7	8	9	10	11	12	13	14	15	16	17	18
年龄																		

18. 威胁要伤害你的兄弟姐妹（包括继兄弟姐妹）。

曾发生	不曾发生

发生	1	2	3	4	5	6	7	8	9	10	11	12	13	14	15	16	17	18
年龄																		

有时不与你住在一起的成年人或比你大的人曾做过伤害你的事情。如果以下行为曾经发生在你的童年（十八岁以前）阶段，请勾选“曾发生”并尽量估计这些伤害行为发生的所有年龄；如果没有发生过，请勾选“不曾发生”。

19. 给你一些不合适的带有性色彩（如性挑逗、性暗示等）的评论或建议。

曾发生	不曾发生

发生	1	2	3	4	5	6	7	8	9	10	11	12	13	14	15	16	17	18
年龄																		

20. 以带有性色彩（如性挑逗、性暗示等）的方式触摸或爱抚你的身体。

曾发生	不曾发生

发生	1	2	3	4	5	6	7	8	9	10	11	12	13	14	15	16	17	18
年龄																		

21. 以带有性色彩（如性挑逗、性暗示等）的方式让你触摸他们的身体。

曾发生	不曾发生

发生	1	2	3	4	5	6	7	8	9	10	11	12	13	14	15	16	17	18
年龄																		

22. 曾试图和你进行口交、肛交、生殖器性交中任一种方式的性行为。

曾发生	不曾发生

发生	1	2	3	4	5	6	7	8	9	10	11	12	13	14	15	16	17	18
年龄																		

有时与你住在一起的父母，继父母或者其他成人（父母的男朋友或女朋友、祖父母）之间会出现激烈的争吵、肢体冲突。如果以下行为曾经发生在你的童年（十八岁以前）阶段，请勾选“曾发生”并尽量估计这些伤害行为发生的所有年龄；如果没有发生过，请勾选“不曾发生”。

23. 看见家里的成年人推、抓、扇你的母亲（继母，祖母）或者朝她扔东西。

曾发生	不曾发生

发生	1	2	3	4	5	6	7	8	9	10	11	12	13	14	15	16	17	18
年龄																		

24. 目睹住在家里的成年人用力打你的母亲（继母、祖母），让她留下的印记或伤痕好几分钟都没有消失。

曾发生	不曾发生

发生年龄	1	2	3	4	5	6	7	8	9	10	11	12	13	14	15	16	17	18

25. 目睹住在家中的成人用力打过你的母亲（或者继母、祖母），或企图通过其他方式伤害她并且使她接受或需要接受药物治疗（吃药、擦药或者去医院等）。

曾发生	不曾发生

发生年龄	1	2	3	4	5	6	7	8	9	10	11	12	13	14	15	16	17	18

26. 看见家里的成年人推、抓、扇你的父亲（继父，祖父）或者朝他扔东西。

曾发生	不曾发生

发生年龄	1	2	3	4	5	6	7	8	9	10	11	12	13	14	15	16	17	18

27. 目睹住在家里的成年人用力打你的父亲（继父、祖父），让他留下的印记或伤痕好几分钟都没有消失。

曾发生	不曾发生

发生年龄	1	2	3	4	5	6	7	8	9	10	11	12	13	14	15	16	17	18

28. 目睹住在家中的成人用力打过你的父亲（或者继父、祖父），或企图通过其他方式伤害他并且使他接受或需要接受药物治疗（吃药、擦药或者去医院等）。

曾发生	不曾发生

发生年龄	1	2	3	4	5	6	7	8	9	10	11	12	13	14	15	16	17	18

有些时候你的同龄人或者比你年长的孩子曾对你做出过欺辱、骚扰等伤害你的事情。如果以下行为曾经发生在你的童年（十八岁以前）阶段，请勾选“曾发生”并尽量估计这些伤害行为发生的所有年龄；如果没有发生过，请勾选“不曾发生”。

29. 每年不止一次诅咒你、指责你，对你爆粗口，对你说“你真胖”、“你真丑”、“你真蠢”等侮辱人的话。

曾发生	不曾发生

发生年龄	1	2	3	4	5	6	7	8	9	10	11	12	13	14	15	16	17	18

30. 每年不止一次说伤害你的话，致使你感到情绪糟糕，窘迫，羞辱。

曾发生	不曾发生

发生年龄	1	2	3	4	5	6	7	8	9	10	11	12	13	14	15	16	17	18

31. 背地里说你坏话、贬损你或散布关于你的谣言。

曾发生	不曾发生

发生年龄	1	2	3	4	5	6	7	8	9	10	11	12	13	14	15	16	17	18

32. 故意在活动或团体中排挤你。

曾发生	不曾发生

发生年龄	1	2	3	4	5	6	7	8	9	10	11	12	13	14	15	16	17	18

33. 以让你害怕会遭受到身体伤害的方式行事。

曾发生	不曾发生

发生年龄	1	2	3	4	5	6	7	8	9	10	11	12	13	14	15	16	17	18

34. 为了得到你的钱或财产而威胁你。

曾发生	不曾发生

发生年龄	1	2	3	4	5	6	7	8	9	10	11	12	13	14	15	16	17	18

35. 曾强迫或威胁你去做你不想做的事情。

曾发生	不曾发生

发生年龄	1	2	3	4	5	6	7	8	9	10	11	12	13	14	15	16	17	18

36. 故意推你、抓你、撞你、扇你耳光、掐你、用拳打你或用脚踢你。

曾发生	不曾发生

发生年龄	1	2	3	4	5	6	7	8	9	10	11	12	13	14	15	16	17	18

37. 很用力地打你，让你留下的印记或伤痕好几分钟都没有消失。

曾发生	不曾发生

发生	1	2	3	4	5	6	7	8	9	10	11	12	13	14	15	16	17	18
年龄																		

38. 曾用力打你，或企图通过其他方式伤害你并使你接受或需要接受药物治疗（吃药、擦药或者去医院等）。

曾发生	不曾发生

发生	1	2	3	4	5	6	7	8	9	10	11	12	13	14	15	16	17	18
年龄																		

39. 强迫你从事一些违背你意愿的性活动。

曾发生	不曾发生

发生	1	2	3	4	5	6	7	8	9	10	11	12	13	14	15	16	17	18
年龄																		

40. 强迫你做一些你不想做的与性相关的事情。

曾发生	不曾发生

发生	1	2	3	4	5	6	7	8	9	10	11	12	13	14	15	16	17	18
年龄																		

如果以下行为曾经发生在你的童年（十八岁以前）阶段，请勾选“曾发生”并尽量估计这些伤害行为发生的所有年龄；如果没有发生过，请勾选“不曾发生”。

41. 你感到你的母亲或者其他重要的母亲角色（如继母、祖母等）的人，虽然会在家中出现但是由于某些原因（如毒品、酒精、过分关注工作、外遇、只顾追求自己的目标等）难以给予你感情支持。

曾发生	不曾发生

发生	1	2	3	4	5	6	7	8	9	10	11	12	13	14	15	16	17	18
年龄																		

42. 你感到你的父亲或者其他重要的父亲角色（如继父、祖父等）的人，虽然会在家中出现但是由于某些原因（如毒品、酒精、过分关注工作、外遇、只顾追求自己的目标等）难以给予你感情支持。

曾发生	不曾发生

发生	1	2	3	4	5	6	7	8	9	10	11	12	13	14	15	16	17	18
年龄																		

43. 你感到你的母亲或其他重要的母亲角色（如继母、祖母等）的人，由于某些原因（如服兵役，照顾一个生病的亲戚，在求学，职业需要）而难以给予你情感支持。

曾发生	不曾发生

发生	1	2	3	4	5	6	7	8	9	10	11	12	13	14	15	16	17	18
年龄																		

44. 你感到你的父亲或其他重要的父亲角色（如继父、祖父等）的人，由于某些原因（如服兵役，照顾一个生病的亲戚，在求学，职业需要）而难以给予你情感支持。

曾发生	不曾发生

发生	1	2	3	4	5	6	7	8	9	10	11	12	13	14	15	16	17	18
年龄																		

曾发生	不曾发生

发生	1	2	3	4	5	6	7	8	9	10	11	12	13	14	15	16	17	18
年龄																		

46. 你认为有一个或多个家庭成员关爱着你。

曾发生	不曾发生

发生	1	2	3	4	5	6	7	8	9	10	11	12	13	14	15	16	17	18

曾发生	不曾发生

发生	1	2	3	4	5	6	7	8	9	10	11	12	13	14	15	16	17	18
年龄																		

48. 有一个或多个家庭成员在你需要的时候带你去看医生或者送你去急症室。

曾发生	不曾发生

发生	1	2	3	4	5	6	7	8	9	10	11	12	13	14	15	16	17	18
年龄																		

49. 有一个或多个家庭成员协助你完成作业或者为你去学校做准备。

曾发生	不曾发生

发生	1	2	3	4	5	6	7	8	9	10	11	12	13	14	15	16	17	18
年龄																		

如果以下事件符合童年（十八岁以前）阶段你和你所在家庭的情况，请勾选 “曾发生” 并尽量估计这些伤害行为发生的所有年龄；如果没有发生过，请勾选 “不曾发生”。

50. 你吃不饱饭。

曾发生	不曾发生

发生年龄	1	2	3	4	5	6	7	8	9	10	11	12	13	14	15	16	17	18

51. 你不得不穿脏的衣服。

曾发生	不曾发生

发生年龄	1	2	3	4	5	6	7	8	9	10	11	12	13	14	15	16	17	18

52. 你在需要被照料的年龄或环境中独自一人。

曾发生	不曾发生

发生年龄	1	2	3	4	5	6	7	8	9	10	11	12	13	14	15	16	17	18

53. 你感到你不得不肩负起成人的责任。

曾发生	不曾发生

发生年龄	1	2	3	4	5	6	7	8	9	10	11	12	13	14	15	16	17	18

54. 你感受到你的家庭处于严重的经济压力之下。

曾发生	不曾发生

发生年龄	1	2	3	4	5	6	7	8	9	10	11	12	13	14	15	16	17	18

55. 你感到一个或者多个人有重大的秘密瞒着你。

曾发生	不曾发生

发生年龄	1	2	3	4	5	6	7	8	9	10	11	12	13	14	15	16	17	18

56. 你的家庭成员互相照顾。

曾发生	不曾发生

发生年龄	1	2	3	4	5	6	7	8	9	10	11	12	13	14	15	16	17	18

57. 你的家庭成员互相感到很亲近。

曾发生	不曾发生

发生年龄	1	2	3	4	5	6	7	8	9	10	11	12	13	14	15	16	17	18

58. 你感到家庭是你力量与支持的源泉。

曾发生	不曾发生

发生年龄	1	2	3	4	5	6	7	8	9	10	11	12	13	14	15	16	17	18
