# Supplementary material for: The Chinese version of the Maltreatment and Abuse Chronology of Exposure (MACE) scale: Psychometric properties in a sample of young adults
Source: PLoS One. 2022 Jun 30;17(6):e0270709. doi: 10.1371/journal.pone.0270709 (PMC9246159; doi:10.1371/journal.pone.0270709)
Supplement: Attachment — Submitted filename: response to reviewer.docx [file pone.0270709.s005.docx]

May 27, 2022

Torsten Klengel, MD PhD

Academic Editor

PLOS ONE

Manuscript Number: PONE-D-22-05087

Dear Dr. Klengel:

Thank you for the constructive feedback from you and the reviewers of our manuscript, “The Chinese version of the Maltreatment and Abuse Chronology of Exposure (MACE) scale: Psychometric properties in a sample of young adults”, Manuscript Number: PONE-D-22-05087. We have revised the manuscript and incorporated all suggestions and comments from you and reviewers. Below are our responses in detail.

**Reviewer #1**

***1. The following sentence is somewhat confusing: “Rasch modeling was used for scale development in a sample of 812 undergraduate students. Item reduction analysis of the original 75 items used to develop the 52-item MACE produced a 58-item Chinese version.” What is the “52-item MACE” that is referred to here? Either add information on what this version is (e.g. it is*** ***the standard US version), or remove the reference to this unidentified version. I would suggest the latter.***

Thanks for your suggestion. We have removed the reference to the unidentified version as follows.

“Item reduction analysis of the original 75 items produced a 58-item Chinese version with ten subdimensions.” (p. 2)

***2. The authors write: “The new scale showed the same factor structure as the original MACE”. It is not clear to me what they mean here, since they did not perform any factor analysis to extract*** ***subdimensions on the MACE. Instead, the authors relied on the 10 existing dimensions of MACE and used Rasch analysis on each of the dimensions to reduce number of items. Indeed, in Methods they write “We aimed to develop ten subscales for the Chinese version of the MACE, corresponding to the ten types of maltreatment assessed by the MACE-X.”. Hence, it appears that the reference to factor structure of the Chinese MACE in the abstract, should be removed or reformulated.***
Thanks for pointing this. To avoid confusion, we removed this sentence.

***3. At the end of the introduction, the authors should specify which psychometric analysis they will perform:*** ***test-retest,*** ***convergent validity, discriminant validity etc.***
True. We specified this at the end of the introduction as follows.

“Rasch modeling, test-retest reliability, convergent validity and predictive validity would be examined.” (p. 6)

***4. Methods section may start out with a paragraph that describes the study design.***

Thanks for pointing this. We described the study design and procedures to the beginning of the ‘Method’ section in revised manuscript. (p. 6)

***5. Part 2.1 on participants: At the end of the paragraph, the authors write that “109 completed all measures again three weeks later to assess test-retest reliability”. I had thought that it was only MACE-CH that was administered again after three weeks, with the other instruments administered only at baseline?***

True. Only the Chinese version of MACE-X was completed again after three weeks. We have modified the description about this. (p. 7)

***6. At the end of the Results section, under the heading “Recollected time of exposure”, the authors present some data on age of exposure. I could not find any notice or description in Methods that such an analysis should be performed or how it can be understood. In results, some aspects of the methods are noted, but this should be moved to Methods. Moreover, it is not fully clear to me (it does not come clearly across) what the authors actually did, and which results they found in terms of ages of exposure – which they present in table 16. Did they find that, for each subdimension, certain ages occurred more often than others?***

Thanks for pointing this. This part is not important and seems redundant. To avoid confusion, we deleted this paragraph in revised manuscript.

**Reviewer #2**

***1.Please add a comment if you plan making the MACE-CH fully available (open access, upon request)***

Thanks for pointing this. The MACE-CH will be provided as open access. We added this point in the ‘Conclusion’ section as follows.

“The MACE-CH will be provided as open access to help facilitate its use.” (p. 24)

***2. The MACE-risk associations are not limited to psychopathology (“identify periods of development when childhood maltreatment maximally increases vulnerability to psychopathology“). Health outcomes?***

True. We modified this description in revised manuscript as follows.

“identify periods of development when childhood maltreatment maximally increases vulnerability to psychopathology and health outcomes” (p. 2)

***3. MACE can also be used in clinical settings and is not only limited to research.***
True. We modified this description in revised manuscript as follows.

“This new instrument will be a valuable tool for Chinese researchers and psychiatrist to ascertain the type and timing of exposure to maltreatment.” (p. 2)

***4. when you refer to the*** ***population-attributable risk. Do you mean the*** ***population-attributional risk fraction?***

Yes. We replaced ‘population-attributable risk’ with ‘population-attributional risk fraction’ in revised manuscript. (p. 3)

***5. Methods: please specify all important parameters that were set e.g. for random effects in*** ***linear mixed effect models.***

Thanks for pointing this. After careful discussion, we deleted the paragraph describing the linear mixed effect models. Thus, none will be changed here.

***6. Please report how you have made sure, that participants reported events correctly (e.g., an event that happened at the age of “5”/”0” needs to be checked for the 6th /first year of life)….***

Thank you. During data collection, we gave subjects the instructions about how to report events correctly. For example, from birth to age one (12 months), they were instructed to tick ‘1’.

***7. Please specify, which items were considered for potential inclusion of each subscales.***

True. These items have been shown in Table 1 to Table 10.

***8. How was the subsample selected that did the retest of the MACE-CH?***

During data collection at baseline, we asked participants whether they could complete the MACE-X again after three months. If they agree, we will invite them to take the retest. We added this information into the ‘Procedure’ section in revised manuscript. (p. 6)

***9. Please explain why Rasch models cannot*** ***export sufficient person parameters? And why*** ***subscale with 4 items were not scored as 0 = 0, 1= 2.5, 2=5, 3 = 7.5 and 4 = 10?***

Based on the number of items positively endorsed by participants and person parameters in the Rasch model, subscales that included 5 or more items were scored for severity of exposure to the latent category. Typically these mean-centered logit scores range from -4 to +4. For the purposes of the current study they were further recalibrated to range from 0–10, enabling total exposure severity levels across the 10 subscales to fall in the range of 0 to 100. In this process, subscales with 4 items could not get mean-centered logit scores due to insufficient item parameters.

We believe that when subscales with 4 items were scored as ‘0 = 0, 1= 2.5, 2=5, 3 = 7.5 and 4 = 10’, the results are similar. In current study, subscales that contained 4 items were scored 0, 3, 5, 8 and 10. This scoring algorithm is aligned with that in the standard US version of MACE.

***10. The age range of the present sample is restricted 18-26years. Would also another slitting would be interesting to detect differential responding for women and men?***

True. We have added the results of Andersen's likelihood ratio tests regarding for women and men in revised manuscript.

*For instance:*

‘When comparing males and females, the Andersen test was also not significant, *χ*^2^(4) = 5.070, *p* = .280.’

***11. The authors seem to consider more strict ranges of infits and outfits for the items as compared to description in the methods (1.3 and 0.7 instead of 1.5 and 0.5). Please be consistent.***

Thanks for pointing this. We have modified the description in revised manuscript.

***12. I would recommend not to mention*** ***the scaled scores*** ***when reporting the number of positively endorsed items for cut off severity (Parental verbal abuse).***

Thanks for this suggestion. We didn’t mention the scaled scores when reporting the number of positively endorsed items for cut off severity in revised manuscript.

***13. Non-verbal emotional abuse: when “******splitting”… instead of “******slipping”.***

Thanks. We have corrected this error.

***14.*** ***Sexual abuse: The df for the Andersen test was 6. Please report which item was dropped for this analysis. And please check the df for other reported tests (e.g., witnessed violence to siblings).***

Thanks for pointing this. There is a clerical error and we have corrected it. We also checked the df for other results.

***15. Please also report*** ***test-retest reliability of the MACE duration (years with a multiplicity score ≥ 1 (ranging from 0 to 18).***

True. We further reported test-retest reliability of the MACE duration (*r* = 0.795, *p* < 10^-16^) in revised manuscript.

***16.*** ***There is an imbalance in number of items per perpetrator.*** ***Only two items of parental sexual abuse were included, while for other adults 4 items were included. This means, that two potentially important items were not scored if endorsed (1) parents attempted to have any type of sexual intercourse with you (2) parents had sexual intercourse with you. From the item difficulties it appears to be rarely reported events. Please revise the subscale or discuss this issue. Would you expect different results when assessing these items*** ***in highly exposed individuals (e.g. individuals with mental illness)?***

True. These two items you mentioned appear to be rarely reported events, contributing to very low Outfit MSQ ( < 0.2 ). Thus, they are not included in our sexual abuse subscale. We expect different results when assessing these items in clinical populations. This point has been mentioned as a limitation in revised manuscript as follow.

“There is an imbalance in number of items per perpetrator within sexual abuse subscale. Only two items of parental sexual abuse were included, while for other adults 4 items were included. Other items of parental sexual abuse appear to be rarely reported events, contributing to very low Outfit MSQ. Thus, they are not included in final subscale of sexual abuse. It would be valuable in future to test whether there are different results when these items of parental sexual abuse are assessed in highly exposed individuals (e.g. individuals with mental illness).”

***17. Table 12: Please*** ***specify the parameter of the MACE that you have used for this correlation.***

Table 12 showed the test-retest reliability for MACE total scores at each age from years 1 to 18. We specified this in revised manuscript.

**Reviewer #3**

***1. The only thing worth correcting would be in the second last paragraph, line 3 of the discussion section, where the*** ***Khan et al paper should be referenced to 2015 and not 2017.***

Sorry for this. We have corrected this error in revised manuscript.

***2. There were only two minor comments, for future considerations.*** ***The duration for testing the test-retest reliability which you have acknowledged as a limitation, was not ideal. Additionally, I wish you had used a scale or two in addition to*** ***the Depression and Anxiety Stress Scales.***

Thanks for pointing this. We added these two points as limitations in revised manuscript as follows.

“Third, the duration for testing the test-retest reliability is not ideal. Future work using a longer duration would be better. Last, in addition to the Depression and Anxiety Stress Scales, more scales rating psychopathology should be used to test the predictive power of MACE-CH.”

We are very grateful to you for the careful and thoughtful feedback you have provided us. Regardless of the final status of our paper, we are confident that it is much better, thanks to your efforts on our behalf.
