# Supplementary material for: The Chinese version of the Maltreatment and Abuse Chronology of Exposure (MACE) scale: Psychometric properties in a sample of young adults
Source: PLoS One. 2022 Jun 30;17(6):e0270709. doi: 10.1371/journal.pone.0270709 (PMC9246159; doi:10.1371/journal.pone.0270709)
Supplement: Attachment — Submitted filename: response to reviewer.docx [file pone.0270709.s006.docx]

Jun 11, 2022

Torsten Klengel, MD PhD

Academic Editor

PLOS ONE

Manuscript Number: PONE-D-22-05087R1

Dear Dr. Klengel:

Thank you for the constructive feedback from you and the reviewers of our manuscript, “The Chinese version of the Maltreatment and Abuse Chronology of Exposure (MACE) scale: Psychometric properties in a sample of young adults”, Manuscript Number: PONE-D-22-05087R1. We have revised the manuscript and incorporated all suggestions and comments from reviewers. Below are our responses in detail.

**Reviewer #1**

***Abstract – last sentence, change “psychiatrist” to “psychiatrists”***

Thank you. We have changed “psychiatrist” to “psychiatrists”. (p. 2)

Introduction
***Page 4, paragraph starting with “Although the..”. Sentence in midst of paragraph, change “in” to “of”, so that sentence reads “This consideration is critically important as there may be periods of development when exposure to specific types of maltreatment is maximally associated with vulnerability for psychopathology and alterations in the structure and function OF stress-susceptible brain regions.”***

Thank you. We have changed ‘in’ to ‘of’. (p. 4)

***Page 5, paragraph starting with “The MACE has shown”, second sentence: Rewrite to something like - “The authors of the MACE have also made available the original set of 75 items, called the MACE-X, from which THEY EXTRA******CTED A 52 ITEM US VERSION OF THE MACE”.***

Thank you. We have modified this sentence according to your advice. (p. 5)

***Page 5, same paragraph as above. In the second last sentence, it is not necessary to once more state what MACE stands for (Maltreatment and…), since this has been done earlier in the paper. Hence, change the sentence to “Thus, this study aimed to (1) establish the MACE-CH, the Chinese version of the MACE scale,..”***

Thank you. We have modified this sentence according to your advice. (p. 5)

Methods

***Please add a paragraph in Methods that succinctly describes items/ content, item scaling, anxiety and depression subscales, and psychometric properties of the Depression Anxiety Stress Scales (DASS) scale (this seems to be lacking).***

True. We provided a succinct description for DASS in revised manuscript as follows. (p. 10)

‘This measure has shown good reliability and validity in prior research [35]. For the purposes of this study, we used the anxiety and depression subscales as the outcome variables. Participants were instructed to assess their anxiety symptoms (e.g., “I was aware of dryness of my mouth”) or depressive symptoms (e.g., “I found it difficult to work up the initiative to do things.”) during the past week. Fourteen items (seven for each) were rated on a four-point scale ranging from 1 (did not apply to me at all) to 4 (applied to me very much of the time). The responses were averaged across items, with higher scores indicating higher anxiety or depressive symptoms.’

***Under the heading “Initial items”, the content of the first sentence has been mentioned before, so this first sentence can be removed/ deleted. Likewise with the second sentence, it can also be removed. Third sentence can then begin the paragraph,with wordings changed to something like “The 75 item MACE-X assesses ten..”***

True. We deleted these two sentences in revised manuscript. (p. 7)

***Under the heading “Evaluation for item inclusion in a subscale”, second paragraph third sentence beginning with “I******nfit depicts” – the word “that” is repeated twice, remove one of them.***

True. We have deleted the redundant term ‘that’. (p. 8)

***Sentence that continues from page 10 to page 11, second part of the sentence: it appears a word or two are missing after the word “the”: “First, ordinary least squares regression was performed to calculate .., using the to ascertain whether MACE had significantly stronger or weaker predictive power than the comparator scale (i.e., QACE-R or CTQ).”***

True. We have modified this sentence in revised manuscript. (p. 11)

Results

***Page 16, subscale for emotional neglect. First sentence, change “the*** ***night” to “the nine”.***

Thanks. We have changed ‘night’ to ‘nine’. (p. 16)

***In the last sentence in Results, page 19, regarding results from multiple regression, the authors write: “Further, v******ariance decomposition analyses found that MACE Severity and Multiplicity scores explained more variance in symptom ratings of anxiety and depression than the CTQ and QACE-R scores did (Table 14 and Table 15).” However, when inspecting the multiple regression parts and variance decomposition parts in Table 15, it does not seem to me that the MACE Multiplicity scores explained more variance than the QACE-R with respect to depressive symptoms: MACE Multi - Beta = 0.100 /p = 0.134 / 2.93% vs QACE-R - Beta = 0.192 / p = 0.004 and 4.17%. Please clarify.***

True. To avoid confusion, we modified this sentence as follows.

“Further, variance decomposition analyses found that MACE Severity scores explained more variance in symptom ratings of both anxiety and depression than the CTQ scores did (Table 14), and MACE Multiplicity scores explained more variance in symptom ratings of anxiety than the QACE-R scores did (Table 15).” (p. 19)

We are very grateful to you for the careful and thoughtful feedback you have provided us. Regardless of the final status of our paper, we are confident that it is much better, thanks to your efforts on our behalf.
